# Time-of-Day Circadian Modulation of Grape-Seed Procyanidin Extract (GSPE) in Hepatic Mitochondrial Dynamics in Cafeteria-Diet-Induced Obese Rats

**DOI:** 10.3390/nu14040774

**Published:** 2022-02-12

**Authors:** Romina M. Rodríguez, Antonio J. Cortés-Espinar, Jorge R. Soliz-Rueda, Christine Feillet-Coudray, François Casas, Marina Colom-Pellicer, Gerard Aragonès, Javier Avila-Román, Begoña Muguerza, Miquel Mulero, Maria Josepa Salvadó

**Affiliations:** 1 Nutrigenomics Research Group, Department of Biochemistry and Biotechnology, Campus Sescelades, Universitat Rovira i Virgili, 43007 Tarragona, Spain; rominamariel.rodriguez@urv.cat (R.M.R.); antoniojesus.cortes@urv.cat (A.J.C.-E.); jorgericardo.soliz@urv.cat (J.R.S.-R.); marina.colom@urv.cat (M.C.-P.); gerard.aragones@urv.cat (G.A.); begona.muguerza@urv.cat (B.M.); mariajosepa.salvado@urv.cat (M.J.S.); 2 Muscle Dynamics and Metabolism (DMEM), National Research Institute for Agriculture, Food and Environment (INRAE), EMN, UMR 866, Université de Montpellier, 34090 Montpellier, France; christine.coudray@inrae.fr (C.F.-C.); francois.casas@inrae.fr (F.C.); 3 Molecular and Applied Pharmacology Group (FARMOLAP), Department of Pharmacology, Faculty of Pharmacy, Reina Mercedes Campus, Universidad de Sevilla, 41012 Sevilla, Spain; javieravila@us.es

**Keywords:** grape-seed procyanidin extract, circadian rhythms, clock genes, Zeitgebers, obesity, nutrition, hepatic metabolism, mitochondrial dynamics

## Abstract

Major susceptibility to alterations in liver function (e.g., hepatic steatosis) in a prone environment due to circadian misalignments represents a common consequence of recent sociobiological behavior (i.e., food excess and sleep deprivation). Natural compounds and, more concisely, polyphenols have been shown as an interesting tool for fighting against metabolic syndrome and related consequences. Furthermore, mitochondria have been identified as an important target for mediation of the health effects of these compounds. Additionally, mitochondrial function and dynamics are strongly regulated in a circadian way. Thus, we wondered whether some of the beneficial effects of grape-seed procyanidin extract (GSPE) on metabolic syndrome could be mediated by a circadian modulation of mitochondrial homeostasis. For this purpose, rats were subjected to “standard”, “cafeteria” and “cafeteria diet + GSPE” treatments (*n* = 4/group) for 9 weeks (the last 4 weeks, GSPE/vehicle) of treatment, administering the extract/vehicle at diurnal or nocturnal times (ZT0 or ZT12). For circadian assessment, one hour after turning the light on (ZT1), animals were sacrificed every 6 h (ZT1, ZT7, ZT13 and ZT19). Interestingly, GSPE was able to restore the rhythm on clock hepatic genes (*Bmal1*, *Per2*, *Cry1*, *Rorα*), as this correction was more evident in nocturnal treatment. Additionally, during nocturnal treatment, an increase in hepatic fusion genes and a decrease in fission genes were observed. Regarding mitochondrial complex activity, there was a strong effect of cafeteria diet at nearly all ZTs, and GSPE was able to restore activity at discrete ZTs, mainly in the diurnal treatment (ZT0). Furthermore, a differential behavior was observed in tricarboxylic acid (TCA) metabolites between GSPE diurnal and nocturnal administration times. Therefore, GSPE may serve as a nutritional preventive strategy in the recovery of hepatic-related metabolic disease by modulating mitochondrial dynamics, which is concomitant to the restoration of the hepatic circadian machinery.

## 1. Introduction

The typical role of mitochondria is oxidative phosphorylation, which provides adenosine triphosphate (ATP) as a primary energy source for most biochemical and physiological processes. However, these intracellular double-membrane-bound structures also play a pivotal role in ion homeostasis, apoptosis, reactive oxygen species (ROS) production, and in several metabolic pathways since they host fatty acid β-oxidation, as well as urea and Krebs cycles [1,2]. Although these organelles were originally considered static cellular powerhouses, it is now known that they also connect and exchange materials with other cellular organelles, including the nucleus and the endoplasmic reticulum [3,4]. In this sense, mitochondrial organelles are now seen as dynamic structures that actively “evolve” in response to the energy demand and supply. Hence, a nutrient-abundant environment is associated with a fragmented mitochondrial network. Meanwhile, a calorie-restricted state tends to elongate mitochondria [5]. These morphological changes exhibit a high plasticity and influence the capacity and efficiency of ATP production in response to changes in energy balance. Thus, in the case of starvation, mitochondrial fusion is needed in order to sustain ATP production and to preserve cell viability [6]. In contrast, mitochondrial fission is related to a decrease in ATP production, an increase in mitochondrial uncoupling and nutrient storage to avoid waste of energy and the deleterious effect of food excess [7]. In mammals, fusion depends on three GTPases: mitofusins 1 and 2 (*Mfn1* and *Mfn2*) for the fusion of the outer mitochondrial membrane (OMM) [8]; and the inner-mitochondrial-membrane (IMM)-located protein optic atrophy 1 (Opa1), which is responsible for IMM fusion [9]. On the other hand, the GTPase dynamin-related protein 1 (*Drp1*), together with OMM-located mitochondrial fission 1 protein (*Fis1*), conducts the fission events. These processes constantly counteract one another; therefore, the inactivation of fission activates fusion and vice-versa [10].

On the other hand, several studies have clearly shown the central role of mitochondrial dysfunction in metabolic diseases, specifically in type 2 diabetes mellitus, obesity, dyslipidemia and nonalcoholic fatty liver disease (NAFLD), as well as in the aging process [11,12,13]. Thus, improper function of mitochondria causes oxidative stress and impaired cellular functions, which could lead to insulin resistance and other metabolic changes typical of metabolic syndrome. Furthermore, it has been described that the reduction in proteins involved in mitochondrial fusion, as well as the increase in proteins in mitochondrial fission, are also characteristic of insulin-resistant states [7,14,15,16]. Additionally, it is well known that the liver plays a central role in glucose homeostasis and is essential for maintaining the body’s overall energetic metabolism. Moreover, it is one of the richest organs regarding density and number of mitochondria. In this sense, most chronic liver diseases are related to mitochondrial malfunction and to the accumulation of damaged mitochondria [17].

Furthermore, metabolism follows circadian rhythms, which are driven by peripheral clocks. The molecular machinery of circadian clock consists of transcriptional and translational feedback loops. The heterodimer formed by circadian locomotor output cycles kaput (CLOCK), and brain and muscle aryl hydrocarbon receptor nuclear translocator-like 1 (*Bmal1*) is the main player in the circadian clock. The CLOCK-*Bmal1* heterodimer binds to E-box, and period (*Per*) and cryptochrome (*Cry*) genes are transcribed. PER and CRY*Per* and *Cry* proteins can heterodimerize to repress the CLOCK-*Bmal1* gene-associated translation. Casein kinase 1 (Ck1) phosphorylates both proteins, PERand CRY, and this step is required to start the repression. Nevertheless, protein phosphatase 1 (Pp1) catalyzes the opposite reaction: the dephosphorylation of these proteins. Post-transcriptional and post-translational processes are able to regulate the PER-CRY repression. CLOCK-*Bmal1* also transcribes retinoid-related orphan receptor (*Ror*) and nuclear receptor subfamily 1 group D (*Rev*-*Erb*). Both products activate and repress respectively, *Bmal1* gene expression [18,19,20,21].

Experimental data support the idea that mitochondrial function is closely related to the clock machinery. In this sense, it has been previously identified that several genes involved in mitochondrial dynamics and mitochondrial respiration are expressed in a daily manner in mouse liver and that these daily oscillations are lost in *Bmal1* liver-specific knockout mice [4]. Moreover, in the absence of *Bmal1*, mitochondria are more vulnerable to damage caused by oxidative stress; additionally, levels of *Mfn1* and *Opa1* fusion proteins are decreased [22]. Using an electron microscope, Uchiyama and collaborators observed significant changes in the shape and volume of mitochondria between the light and dark cycle in hepatocytes of Wistar rats [23].

Additionally, mitochondrial dynamics might also influence circadian rhythms. Studies carried out using *Drp1*-deficient mice showed an important loss of *Bmal1* and *Per1/2* circadian oscillations, as well as a loss of circadian ATP production. This evidence suggest that circadian activation of *Drp1* plays an important role in connecting circadian and mitochondrial metabolic cycles [24].

A high-fat diet induces a delay in the liver circadian-clock machinery, which promotes the activation of hepatic stellate cells and damage in hepatocytes, causing hepatic steatosis, in some cases leading to cell death. All these events result in a disruption of the normal circadian rhythm and a decrease in the amplitude of circadian-clock oscillations [25,26,27].

Polyphenols are secondary compounds widely distributed in the plant kingdom. There is evidence that phenolic substances act as antioxidants by preventing the oxidation of low-density lipoprotein (LDL), platelet aggregation, and damage of red blood cells [28]. Additionally, phenolics act as (i) metal chelators, (ii) antimutagens and anticarcinogens, (iii) antimicrobial agents and (iv) anti-inflammatory agents [29].

Interestingly, polyphenols have been shown to restore specific disruptions in the circadian clock due to nutrient overload [30,31,32]. In this regard, it has been found that dietary tea polyphenols ameliorate metabolic syndrome via circadian-clock-related mechanisms [33]. Interestingly, it has also been shown that polyphenol pretreatment ameliorated hydrogen-peroxide (H_2_O_2_)-elicited mitochondria impairment in a *Bmal1*-dependent manner and that such a *Bmal1*-dependent effect was involved in tea polyphenol-stimulated *Nrf2/Are/Ho-1* and *Akt/Creb/Bsnf* signaling pathway [34]. Additionally, Pajuelo and collaborators [35,36] demonstrated the beneficial effects of supplementation of a grape-seed proanthocyanidin extract (GSPE) on mitochondrial dysfunction of brown adipose tissue and skeletal muscle caused by an obesity-induced diet in rats.

Considering these previous considerations, the aim of the present study was to evaluate the possible misalignment of hepatic circadian rhythm in relationship with mitochondrial function caused by an obesity-induced cafeteria diet and to assess whether GSPE supplementation in these obese rats could mitigate expected detrimental effects.

## 2. Materials and Methods

### 2.1. Experimental Procedure in Animals

Ninety-six 12-week-old male Fischer 344 rats (Charles River Laboratories, Barcelona, Spain) were used in this experiment. Rats were housed in pairs at 22 °C, 55% humidity and under a standard photoperiod of 12 h of light and 12 of darkness. A 4-day adaptation period was carried out where rats were fed with a standard diet (STD) ad libitum. The STD composition was 20% protein, 8% fat and 72% carbohydrates (Panlab, Barcelona, Spain). Rats were randomly divided into 2 groups, depending on the diet; 32 rats were fed with STD, and 64 rats were fed a cafeteria diet (CAF) during a 5-week pre-treatment. CAF consisted of biscuits with cheese and pâté, bacon, coiled puff pastry from Mallorca (Hacendado, Spain), feed, carrots and sweetened milk (22% sucrose *w*/v). CAF composition was 14% protein, 35% fat and 76% carbohydrates. The treatment period started at the 5th week and lasted for 4 weeks. Rats continued with the diet they were fed during the pre-treatment period. The administration of treatments was performed at two time points: 48 rats were treated at the beginning of the light phase (8 a.m., ZT0), and 48 rats were treated at the beginning of the dusk phase (8 p.m., ZT12). All STD-fed rats were treated with condensed milk or vehicle (VH). CAF-feed rats were divided into two groups; 32 were treated with the VH, and 32 with 25 mg/kg GSPE (Les Dérivés Résiniques et Terpéniques, Dax, France) diluted 1/5 in condensed milk. GSPE was composed of catechin (58 μmol/g), dimeric procyanidins (250 μmol/g), epicatechin (52 μmol/g), epigallocatechin (5.50 μmol/g), epicatechin gallate (89 μmol/g), epigallocatechin gallate (1.40 μmol/g), hexameric procyanidins (0.38 μmol/g), pentameric procyanidins (0.73 μmol/g), tetrameric procyanidins (8.8 μmol/g) and trimeric procyanidins (1568 μmol/g) [37]. The treatment was orally administered daily using a syringe. During both the pre-treatment and treatment, body weight and food intake were recorded weekly. The rats were fasted for 3 h, then sacrificed by decapitation. Each diet-treatment group was divided into 4 sub-groups of 4 rats, depending on the time of sacrifice (9 a.m. (ZT1), 3 p.m. (ZT7), 9 p.m. (ZT13) or 3 a.m. (ZT19)). Blood was collected to obtain serum after a 15-min centrifugation (12,000× *g* and 4 °C) and stored at −80 °C. The liver was collected and stored at −80 °C for further analysis. All animal care and experimental protocols with animals were approved by the Ethics Review Committee for Animal Experimentation of the Universitat Rovira i Virgili (reference number 9495, 18 September 2019) and were carried out in accordance with Directive 86/609EEC of the Council of the European Union and the procedure established by the Departament d’Agricultura, Ramaderia i Pesca of the Generalitat de Catalunya.

### 2.2. RNA Extraction

A liver-tissue portion (20–30 mg) was mixed with Trizol^®^ reagent (Thermo Fisher, Madrid, Spain) and homogenized by Tissue Lyser LT (Qiagen, Madrid, Spain). After a 10-min centrifugation (12,000× *g* and 4 °C), the homogenate was placed into a new eppendorf tube, and 120 μL of chloroform was added. Two phases were separated after a 15-min centrifugation (12,000× *g* and 4 °C). The aqueous phase was transferred into a new eppendorf tube, and 300 μL of isopropanol was added. After an overnight incubation at −20 °C, samples were centrifugated at 4 °C and 12,000×*g* for 10 min. The supernatant was discarded, and the pellet was cleaned twice with 500 μL and centrifuged for 5 min (8000× *g* and 4 °C). The supernatant was discarded again, and the washed pellet was resuspended with 60 μL of nuclease-free water (Thermo Fisher, Madrid, Spain). RNA concentration (ng/μL) and purity were measured by a Nanodrop ND-1000 spectrophotometer (Thermo Fisher, Madrid, Spain).

### 2.3. Gene-Expression Analysis

Complementary deoxyribonucleic acid (cDNA) was obtained by a reverse transcription of the RNA extracted using a high-capacity complementary DNA reverse-transcription kit (Thermo Fisher, Madrid, Spain). Quantitative polymerase chain reactions (qPCRs) were performed in 384-well plates in a 7900HT fast real-time PCR (Thermo Fisher, Madrid, Spain) using iTaq™ Universal SYBR^®^ Green Supermix (Bio-Rad, Barcelona, Spain). The thermal-cycle program used in all qPCRs was 30 s at 90 °C, 40 cycles of 15 s at 95 °C and 1 min at 60 °C. The analyzed liver genes were normalized by the housekeeping gene peptidylprolyl isomerase A (Ppia). The primers used for each gene were obtained from Biomers (Ulm, Germany) and can be found in Table 1. The relative expression of each gene was calculated using the 2^-∆∆Ct^ method, as reported by Schmittgen and Livak [38].

### 2.4. Serum Analysis

Enzymatic colorimetric assays were used for the analysis of glucose, total cholesterol (TC), triglycerides (TAG) (QCA, Amposta, Tarragona, Spain) and non-esterified free fatty acids (NEFAs) (WAKO, Neuss, Germany) according to the manufacturer’s instructions.

### 2.5. Extraction and Measurement of Concentrations of Lipids in Liver

Liver lipids were extracted following the Bligh–Dyer method [39], and levels of hepatic cholesterol, TAG and total lipid liver content were measured using a colorimetric kit assay (QCA, Barcelona, Spain).

### 2.6. Determination of Liver Mitochondrial Enzymatic Activities

Liver samples were homogenized in a cold phosphate buffer (50 mM, pH 7) in the following proportion: 0.5 g of liver tissue for 4.5 mL buffer using a Polytron homogenizer (Kinematica, Switzerland). Then, homogenates were centrifuged at 1000× *g* for 10 min at 4 °C, and supernatants were collected for enzymatic analysis. Citrate synthase (CS) activity was determined as described by Srere [40]: the deacetylation of acetyl coenzyme A (Acetyl-CoA) in the presence of oxalacetate and 5,5′-dithiobis-2-nitrobenzoic acid (DNTB) produced by CS can be followed spectrophotometrically at 412 nm for 30 s due to the formation of 5-thio-2-nitrobenzoic acid (TNB). Complex I (CI) activity was determined as described by Janssen et al. [41], spectrophotometrically measuring, at 600 nm for 30 s, the reduction of 2,6-dichloroindophenol (DCPIP) produced by the electrons accepted from decylubiquinol, reduced by complex I in a previous step. The activity of Complex II (CII) and complex II + III (CII + III) was measured according to Rustin et al. [42], spectrophotometrically following the reduction of DCPIP by the succinate at 600 nm for 1 min for CII and the oxidation of cytochrome c at 550 nm for 1 min for CII + III. Cytochrome c oxidase (COX) activity was determined as described by Wharton and Tzagoloff [43], spectrophotometrically measuring oxidation of cytochrome c reduced at 550 nm for 30 s. 

### 2.7. Metabolomic Analysis

Metabolomic analysis of the 96 rat liver samples was performed at the Centre for Omic Sciences (COS, Tarragona, Spain) using gas chromatography coupled with quadrupole time-of-flight mass spectrometry (GC-qTOF model 7200, Agilent, Santa Clara, CA, USA). The extraction was performed by adding 400 µL of methanol:water (8:2)-containing internal standard mixture to liver samples (approx. 10—20 mg). Then, the samples were mixed and homogenized on a bullet blender using a stainless-steel ball, incubated at 4 °C for 10 min and centrifuged at 19,000× *g* rpm; supernatant was evaporated to dryness before compound derivatization (methoximation and silylation). The derivatized compounds were analyzed by GC-qTOF. Chromatographic separation was based on the Fiehn Method, [44] using a J&W Scientific HP5-MS film capillary column (30 m × 0.25 mm × 0.25 µm, Agilent, Santa Clara, CA, USA) and helium as carrier gas with an oven program from 60 to 325 °C. Ionization was done by electronic impact (EI), with electron energy of 70 eV and operating in full-scan mode. Identification of metabolites was performed using commercial standards and by matching their EI mass spectrum and retention time to a metabolomic Fiehn library (from Agilent, Santa Clara, CA, USA), which contains more than 1400 metabolites. After putative identification of metabolites, they were semi-quantified in terms of internal standard response ratio.

### 2.8. Circadian-Rhythm Analysis

To analyze the circadian rhythms of the different clock genes, we used a cosinor-based rhythmometry method. We considered the presence of circadian rhythm when the model of each gene expressions fit the cosine curves (*p* < 0.05). For this, a script was developed by J.R. S.-R. using PyCharm software (v. 2018.2.4, JetBrains s.r.o., Prague, Czech Republic) with Python Software Foundation version 3.7.4 (Wilmington, DE, USA), and circadian-rhythm estimates were plotted using CosinorPy package (v. 1.1) (Ljubljana, Slovenia) [45].

### 2.9. Statistical Analysis

Data are reported as mean ± standard error of the mean (SEM). Biometric parameters, liver mitochondrial enzymatic activity, liver weight and liver gene expression were subjected to Student’s t test, as well as one- and two-way analysis of variance (ANOVA) with the least significant difference test (LDS) for post hoc comparisons using the computer program SPSS version 25 (SPSS Inc., Chicago, IL, USA). Graphics were done by GraphPad Prism 8 software (San Diego, CA, USA). For all analyses, a probability (*p*) value of <0.05 was considered statistically significant. 

Heatmaps of metabolites were performed after data normalization and autoscaling using MetaboAnalyst 5.0 software (Edmonton, Alberta, Canada; https://www.metaboanalyst.ca/, accessed on 20 December 2021) [46].

## 3. Results

### 3.1. Animal Body-Weight Gain Corroborates the Obesogenic Effect of the Cafeteria Diet

**Figure 1:**
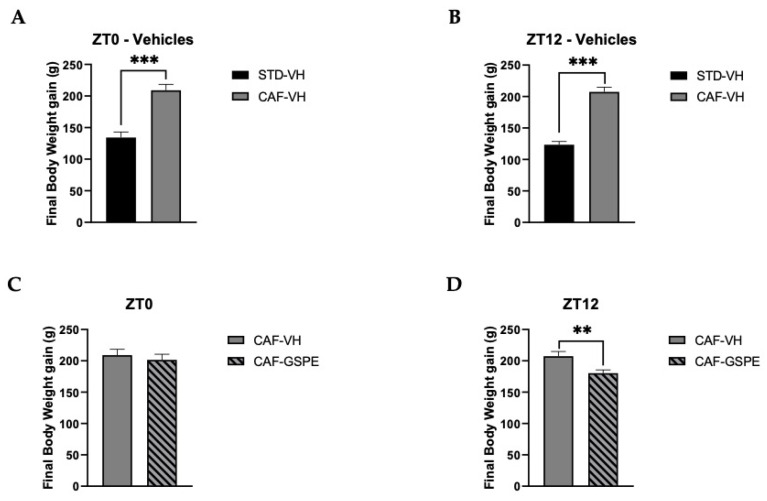
Body-weight gain (g). (**A**) Body-weight gain in grams of ZT0-vehicle rats (standard diet-vehicle (STD-VH) and cafeteria-diet-vehicle rats (CAF-VH) in the nine weeks of the experiment. (**B**) Body-weight gain in grams of ZT12-vehicle rats (STD-VH and CAF-VH) in the nine weeks of the experiment. (**C**) Body-weight gain in grams of ZT0-CAF-VH and cafeteria diet–grape-seed proanthocyanidin extract (CAF-GSPE) groups in the nine weeks of the experiment. (**D**) Body-weight gain in grams of ZT12 CAF-VH and CAF-GSPE groups in the nine weeks of the experiment. *** Indicates significant differences using repeatedly measured ANOVA followed by Student’s *t* test between VH groups (STD-VH vs. CAF-VH) (*p* ≤ 0.001); ** indicates significant differences within CAF groups (CAF-VH vs. CAF-GSPE) (*p* ≤ 0.01).

The final body-weight gain of animals fed with CAF diet was significantly higher than that of animals fed an STD diet (*p* = 0.0001) (Figure 1A,B). After demonstrating the efficacy of the CAF diet in the experimental animals, the different treatments were analyzed. In this sense, Figure 1C,D shows the comparison of body-weight gain between the two treatments—VH or GSPE—in the morning (ZT0) and at night (ZT12) in rats fed a CAF diet. Interestingly, animals treated with GSPE at night (ZT12) showed a reduction in body-weight gain when compared to the control CAF-fed group (*p* = 0.005). This result points out a first differential effect of GSPE depending on the time of administration. Furthermore, globally, this result is in agreement with previous studies that showed GSPE efficiency for treating metabolic syndrome and, more specifically, body weight [47,48,49]. During the ZT12 treatment, rats treated with GSPE consumed fewer kilojoules (kJ) than rats treated with VH (732.5 ± 22.38 vs. 674.9 ± 14.77 kJ; *p* = 0.03). These results are in concordance with the inhibition effects of GSPE over food intake described by Serrano and colleagues [50]. Nevertheless, these differences were not seen in the ZT0 treatment.

### 3.2. Intake of Obesogenic Diet Alters Circulating Levels of Glucose, TAG and TC

Fasting serum glucose, TC, TAG and NEFAs levels were measured after animals were sacrificed at four different circadian time points (ZT1 to ZT19). On the one hand, regarding morning treatments (ZT0), glucose levels in the STD-VH group remained constant at almost all circadian points, except for the last one (ZT19), where its concentration was decreased (Table 2). Moreover, it could also be observed that glucose levels of the CAF-VH group were significantly higher than those of the control group at the ZT7 (*p* = 0.007) and ZT13 (*p* = 0.021) time points, possibly due to the hyperglycemic state associated with obesity. When comparing between the CAF-VH and CAF-GSPE groups, no significant differences in glucose levels were observed. However, GSPE supplementation in the CAF diet tended to improve glucose levels compared to the CAF-VH group at ZT13 (*p* = 0.08). It is noteworthy that at this time point, the CAF-GSPE group reached a similar glucose level to that of the control group. On the other hand, regarding night treatment (ZT12), glucose levels of the STD-VH group remained constant from ZT1 to ZT19, and CAF promoted a homeostatic disruption by increasing glucose levels around 40 percent at all time points. Even though no significant differences were observed between CAF-VH and GSPE groups, serum levels of animals treated with GSPE at ZT13 and ZT19 tended to decrease.

The serum lipid profile was analyzed by TAG and TC levels in all rats after sacrifice and is presented in Table 2 for each time of death. Among the treatments, the CAF-VH showed the highest levels of TAG at each time of death. In the case of diurnal treatment (ZT0), the CAF-GSPE group presented a slight decrease in TAG levels at all time points compared to CAF-VH-treated rats, showing the highest reductions of, 12 and 33%, at ZT7 and ZT13, respectively. These differences were not observed at ZT12, where the levels of TAG in CAF-VH and GSPE groups were similar. The cholesterol levels did not show such an apparent oscillatory pattern as was present for TAG. However, there was a peak in its level at ZT7 in the CAF-VH group treated in the morning (ZT0) and at ZT19 in the CAF-VH nocturnally treated animals, increasing 33 and 44 percent in comparison with the STD-VH group. Regarding NEFA plasmatic levels, no significant differences were found in any experimental group comparison (STD-VH versus CAF-VH and CAF-VH versus CAF-GSPE). 

### 3.3. GSPE Restores CAF Diet-Related Disruption of Circadian Rhythm of Liver Core-Clock Genes 

To evaluate the effects of diet and GSPE treatments on circadian rhythm of the peripheral-clock genes in the liver, we used the cosinor method to model the oscillation of these genes in a 24 h period. This method allowed for the assessment of different circadian parameters, such as amplitude, acrophase and midline estimating statistic of rhythm (MESOR), as well as the comparison of these oscillations and parameters between the different treatment groups (Supplementary Materials, Table S1).

**Figure 2:**
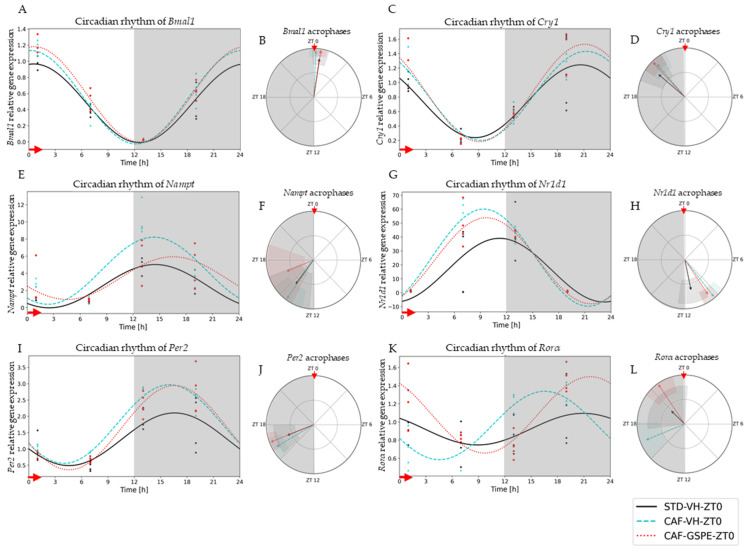
Estimated circadian rhythms (relative gene expression) of groups treated in the morning (ZT0). (**A**) Estimated circadian rhythms and (**B**) acrophases with their amplitudes represented of *Bmal1* for ZT0-vehicle and treatments groups (STD-VH, CAF-VH and CAF-GSPE). (**C**) Estimated circadian rhythms and (**D**) acrophases with their amplitudes represented of *Cry1* for ZT0-vehicles and treatments groups (STD-VH, CAF-VH and CAF-GSPE). (**E**) Estimated circadian rhythms and (**F**) acrophases with their amplitudes represented of *Nampt* for ZT0-vehicle and treatments groups (STD-VH, CAF-VH and CAF-GSPE). (**G**) Estimated circadian rhythms and (**H**) acrophases with their amplitudes represented of Nr1d1 for ZT0-vehicle and treatments groups (STD-VH, CAF-VH and CAF-GSPE). (**I**) Estimated circadian rhythms and (**J**) acrophases with their amplitudes represented of *Per2* for ZT0-vehicle and treatments groups (STD-VH, CAF-VH and CAF-GSPE). (**K**) Estimated circadian rhythms and (**L**) acrophases with their amplitudes represented of *Rorα* for ZT0-vehicles and treatments groups (STD-VH, CAF-VH and CAF-GSPE).

**Figure 3:**
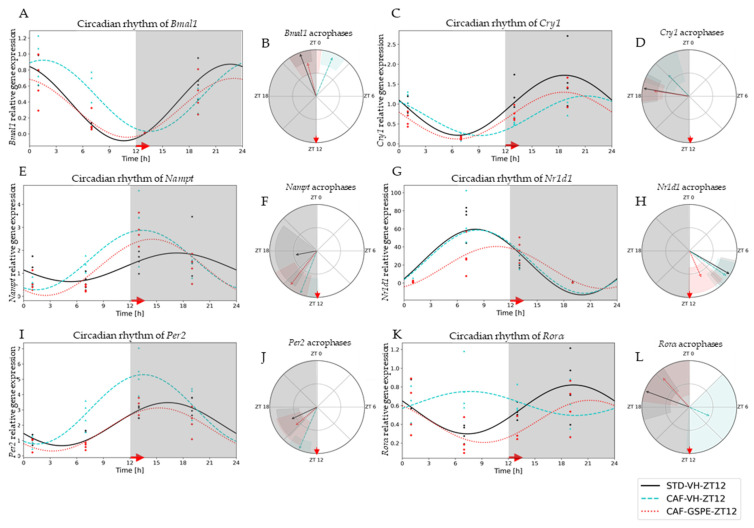
Estimated circadian rhythms (relative gene expression) of groups treated at night (ZT12). (**A**) Estimated circadian rhythms and (**B**) acrophases with their amplitudes represented of *Bmal1* for ZT12-Vehicles and Treatments Groups (STD-VH, CAF-VH and CAF-GSPE). (**C**) Estimated circadian rhythms and (**D**) acrophases with their amplitudes represented of *Cry1* for ZT12-Vehicles and Treatments Groups (STD-VH, CAF-VH and CAF-GSPE). (**E**) Estimated circadian rhythms and (**F**) acrophases with their amplitudes represented of *Nampt* for ZT12-Vehicles and Treatments Groups (STD-VH, CAF-VH and CAF-GSPE). (**G**) Estimated circadian rhythms and (**H**) acrophases with their amplitudes represented of Nr1d1 for ZT12-Vehicles and Treatments Groups (STD-VH, CAF-VH and CAF-GSPE). (**I**) Estimated circadian rhythms and (**J**) acrophases with their amplitudes represented of *Per2* for ZT12-Vehicles and Treatments Groups (STD-VH, CAF-VH and CAF-GSPE). (**K**) Estimated circadian rhythms and (**L**) acrophases with their amplitudes represented of *Rorα* for ZT12-Vehicles and Treatments Groups (STD-VH, CAF-VH and CAF-GSPE).

Interestingly, it was observed that the expression of clock genes presented a clear and robust circadian rhythm in practically all the experimental groups (Figure 2 and Figure 3). However, a clear disruption caused by CAF diet was detected in some parameters, such as amplitude (*p* = 0.047) and acrophase (*p* = 0.033) of N1rd1 gene expression in the groups treated in the morning (ZT0) (Figure 2G,H). In this case, a significant displacement of the acrophase was observed, occurring before the moment of the greatest expression of this gene regarding the standard group. In addition, an increase in its expression was observed at that time point compared to its STD-diet group, increasing the amplitude of oscillation (Figure 2H). Another gene affected by CAF diet was *Rorα*; in this case, a strong displacement of the acrophase in the CAF group in comparison to the control-diet group was clearly observed (*p* = 0.022) (Figure 2K,L). GSPE treatment was able to reverse this acrophase shift in *Rorα*, returning the time point of the highest expression of this gene to similar values as those of the control group. For these groups treated in the morning (ZT0), no other significant difference was detected.

Regarding the night-treated groups (ZT12), CAF-diet effects were detected in genes such as *Bmal1*, *Per2*, *Cry1*, *Rorα* and *Nampt*. The CAF-diet effect on *Bmal1* expression resulted in an acrophase displacement in comparison to the STD-diet group (*p* = 0.0001) (Figure 3A,B). This displacement was significantly restored by GSPE treatment (*p* = 0.0001) (Supplementary Materials, Table S2). Another gene also altered by CAF diet was *Per2* (Figure 3I,J). A similar situation was detected for *Cry1* gene expression; its acrophase was displaced with the CAF diet (*p* = 0.036) and restored with the GSPE treatment at ZT12 (*p* = 0.001) (Figure 3C,D). In this case, a significant displacement of acrophase and an increase in amplitude with CAF diet was observed; nevertheless, the administration of GSPE restored both circadian parameters. Interestingly, the most altered gene was *Rorα*, the circadian rhythmic expression of which was fully disrupted (*p* = 0.037) by the CAF diet (Figure 3K,L). This total disruption of rhythmicity was significantly restored by GSPE treatment (*p* = 0.046), and both circadian parameters (acrophase and amplitude) were reestablished to similar values as those in the STD-diet group (*p* = 0.000). Finally, circadian rhythm of *Nampt* expression was also altered by the CAF diet, shifting its acrophase (*p* = 0.033). Intriguingly, in this case, GSPE treatment was not able to restore its circadian disruption.

### 3.4. GSPE Modulates Changes in Mitochondrial Dynamics and Biogenesis Induced by Cafeteria Diet

**Figure 4:**
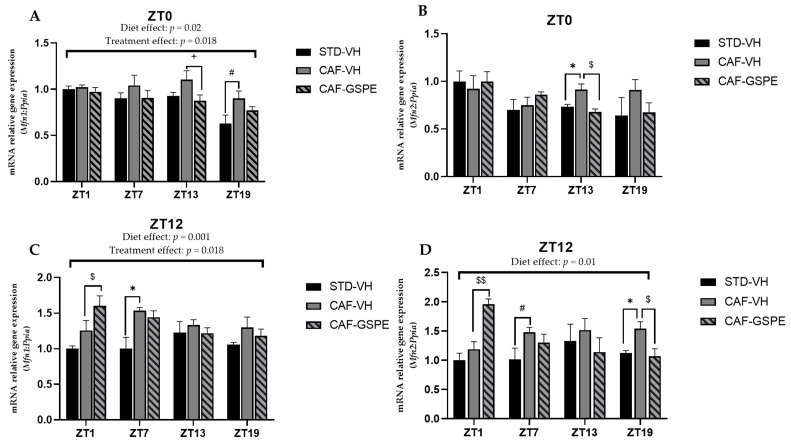
Relative gene expression of mitochondrial fusion genes in the liver. Rats were fed an STD or CAF diet and received a daily dosage of vehicle or GSPE in the morning (ZT0) (**A**,**B**) or at night (ZT12) (**C**,**D**). After 9 weeks, the rats were sacrificed at 9 a.m. (ZT1), 3 p.m. (ZT7), 9 p.m. (ZT13) or 3 a.m. (ZT19), and mRNA levels of *Mfn1* and *Mfn2* were determined. The values are the mean ± SEM (*n* = 4). * The effect of diet within vehicle groups (Student’s *t* test or DMS post hoc test, *p* < 0.05); $ the effect of GSPE consumption within CAF groups (Student’s *t* test or DMS post hoc test, $ *p* ≤ 0.05, $$ *p* ≤ 0.01); # indicates tendency between STD-VH and CAF-VH using Student’s *t* test (*p* = 0.1–0.051); + indicates tendency between CAF-VH and CAF-GSPE using Student’s *t* test (*p* = 0.1–0.051).

**Figure 5:**
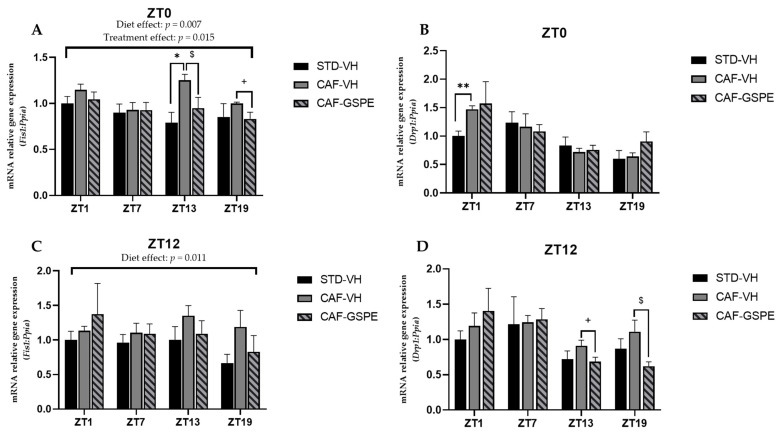
Relative gene expression of mitochondrial fission genes in the liver. Rats were fed an STD or CAF diet and received a daily dosage of vehicle or GSPE in the morning (ZT0) (**A**,**B**) or at night (ZT12) (**C**,**D**). After 9, weeks the rats were sacrificed at 9 a.m. (ZT1), 3 p.m. (ZT7), 9 p.m. (ZT13) or 3 a.m. (ZT19), and mRNA levels of *Fis1* and *Drp1* were determined. The values are the mean ± SEM (*n* = 4). * The effect of diet within vehicle groups (Student’s *t* test or DMS post hoc test, * *p* ≤ 0.05, ** *p* ≤ 0.01); $ the effect of GSPE consumption within CAF groups (Student’s *t* test or DMS post hoc test, *p* < 0.05; + indicates tendency between CAF-VH and CAF-GSPE using Student’s *t* test (*p* = 0.1–0.051).

**Figure 6:**
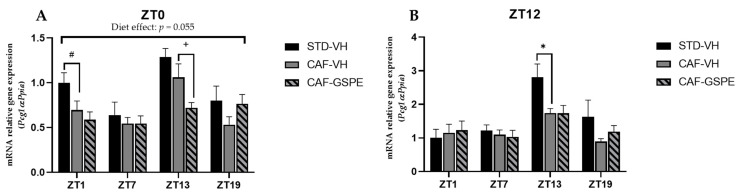
Relative gene expression of mitochondrial biogenesis gen in the liver. Rats were fed an STD or CAF diet and received a daily dosage of vehicle or GSPE in the morning (ZT0) (**A**) or at night (ZT12) (**B**). After 9 weeks, the rats were sacrificed at 9 a.m. (ZT1), 3 p.m. (ZT7), 9 p.m. (ZT13) or 3 a.m. (ZT19), and mRNA levels of *Pgc1α* were determined. The values are the mean ± SEM (*n* = 4). * The effect of diet within vehicle groups (Student’s *t* test or DMS post-hoc test, *p* < 0.05); # indicates tendency between STD-VH and CAF-VH using Student’s *t* test (*p* = 0.1–0.051); + indicates tendency between CAF-VH and CAF-GSPE using Student’s *t* test (*p* = 0.1–0.051).

Several studies suggest that mitochondrial dysfunction plays a critical role in initiating or propagating events in fatty liver disfunction and NAFLD [51]. Moreover, mitochondrial dysfunction and obesity are tightly connected, as these organelles embrace fatty acid oxidation. It should be noted that β-oxidation depends on organelle activity and number to efficiently synthesize ATP from oxidation of metabolic fuels [52]. Therefore, to evaluate mitochondrial dynamics and biogenesis in the liver, gene-expression assessment of mitochondrial fusion (*Mfn1* and *Mfn2*), fission (*Drp1* and *Fis1*) and biogenesis (*Pgc1a*) at each time of death condition (ZT1 to ZT19) was performed (Figure 4, Figure 5 and Figure 6).

As shown in Figure 4, at ZT13 and ZT19, there is a slight effect of GSPE in the morning treatment (ZT0) on *Mfn1* (*p* = 0.96) (Figure 4A) and *Mfn2* (*p* = 0.01) (Figure 4B), avoiding the increase in these gene expressions induced by cafeteria treatment. This effect is also evident in Mnf2 expression in the nocturnal treatment (ZT12) at the ZT19 death time (*p* = 0.03) (Figure 4D). Thus, in relation to mitofusin expression, and except for this last ZT19 condition, the night GSPE treatment behaves completely differently than for the rest of ZTs in comparison with the day GSPE treatment. The most remarkable result is that there is a clear overexpression in both *Mnf1* and *Mfn2*, a 27 and 65% increment, respectively, in the GSPE group in the night treatment (ZT12) compared with the CAF-VH group. Altogether, these results could suggest that the effect of GSPE inducing the fusion of mitochondria seems to be more relevant with night treatment and shortly after GSPE ingestion (ZT1) in cafeteria-fed animals.

Regarding fission genes, when comparing VH groups (CAF vs. STD) treated in the morning (ZT0), it can be seen that CAF-fed animals exhibit significantly higher expression levels of *Drp1* and *Fis1* at ZT1 (*p* = 0.011) and ZT13 (*p* = 0.004), respectively. These differences were not observed between STD-VH and CAF-GSPE groups (Figure 5A–B). Regarding the GSPE effect, a decrease of almost 30 percent in *Fis1* gene expression is observed in the ZT13 condition. As some of these effects were also observed for the fusion genes, it seems that morning GSPE treatment promotes a relatively mild effect and prompts an equilibrium between the fusion and fission events, suggesting that there could be a balance in the morphology of the mitochondrial population.

On the other hand, concerning the expression of the fission genes in the GSPE night treatment (Figure 5C–D), we found significant effects at ZT13 (*p* = 0.069) and ZT19 (*p* = 0.032) in *Drp1* gene expression (between CAF-VH and CAF-GSPE group). In consequence, it seems that in the night treatment, mitochondria could be more present in an elongated/fussed form due to both the induction of fusion genes and the inhibition of fission genes induced by GSPE. 

Mitochondrial biogenesis is regulated by transcription factor *Pgc1α*. As shown in Figure 6A,B, *Pgc1α* expression of STD-VH animals is three-fold higher at ZT13 in both diurnal and nocturnal treatments, as well as at ZT1 in the morning treatment (ZT0) (*p* = 0.090). Interestingly, there is a subtle effect (tendency) of GSPE to decrease biogenesis in the morning treatment at ZT13 (*p* = 0.076). Nevertheless, it seems that GSPE is more capable of modulating the fission and fusion events than biogenesis events. It should be noted that while mitochondrial dynamics are highly regulated by a few genes (*Mfn1/2*, *Drp1*, and *Fis1*), mitochondrial biogenesis involves more factors, which is why, perhaps, no effect of GSPE treatment on the master regulator *Pgc1-α* is observed. Thus, it is likely that other genes, such as *Tfam*, could be also acting. 

### 3.5. Mitochondrial Respiratory Activity Is Highly Altered Due to CAF Diet but Partially Ameliorated by GSPE 

**Figure 7:**
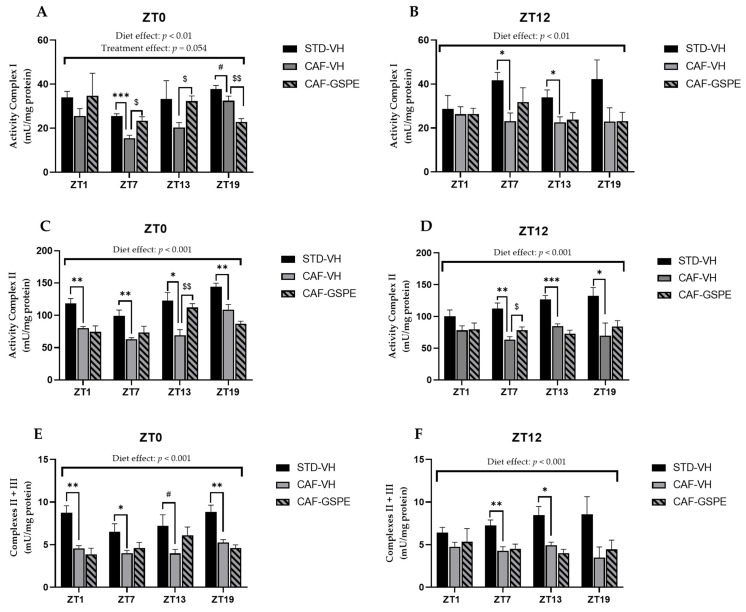
Mitochondrial respiratory activity in the liver. Rats were fed an STD or CAF diet and received a daily dosage of vehicle or GSPE in the morning (ZT0) (**A**,**C**,**E**) or at night (ZT12) (**B**,**D**,**F**). After 9 weeks, the rats were sacrificed at 9 a.m. (ZT1), 3 p.m. (ZT7), 9 p.m. (ZT13) or 3 a.m. (ZT19), and the activity of mitochondrial complexes I, II and III was determined. The values are the mean ± SEM (*n* = 4). * The effect of diet within vehicle groups (Student’s *t* test or DMS post hoc test, * *p* ≤ 0.05, ** *p* ≤ 0.01, *** *p* ≤ 0.001); $ the effect of GSPE consumption within CAF groups (Student’s *t* test or DMS post hoc test, $ *p* ≤ 0.05, $$ *p* ≤ 0.01); # indicates tendency between STD-VH and CAF-VH using Student’s *t* test (*p* = 0.1–0.051).

As it is well known, mitochondrial activity dysfunction is highly related to steatosis and steatohepatitis, as well as fibrosis [53]. For this reason, we measured the activity of the complexes that form part of the respiratory chain. In this sense, as can be seen in Figure 7, a clear alteration produced by CAF diet regarding mitochondrial complexes was observed. Particularly, the activity of mitochondrial complexes I, II and II + III were altered at different times of day.

Regarding complex I, there was a disruption in its activity due to the CAF diet when CAF-VH groups were compared with STD-VH groups, as shown in Figure 7A,B. In addition, these differences were increased when the activity of complex I was measured at ZT7, regardless of the treatment time (*p* = 0.0007 at ZT0 and *p* = 0.012 at ZT12). However, when GSPE was supplied in the morning (ZT0), there was an improvement of the activity at ZT7 (*p* = 0.013) and at ZT13 (*p* = 0.010), with values like those of the STD group. Furthermore, diurnal GSPE-treated animals (ZT0) showed a tendency to increase complex I activity (*p* = 0.054) when compared to treated and non-treated CAF groups (Figure 7A). 

With respect to complex II, the activity of the complex was extremely altered due to the CAF diet, as shown in Figure 7C,D, with activity disruptions at all measured times, except for ZT1, when treated at night (ZT12). Nevertheless, when GSPE treatment was supplied in the morning (ZT0), there was an amelioration of the activity of this complex at ZT13 (*p* = 0.007), showing an activity similar to that in the STD-fed group, which could suggest that GSPE improved the state of this complex. 

As expected, the activity of complexes II + III was also highly altered due to the CAF diet, showing a decrease in activity of almost 50% in some of the cases compared to the STD-fed group, as shown in Figure 7E,F. In addition, this decrease in activity was accentuated at ZT1 when VH was supplied in the morning (ZT0) (*p* = 0.003). However, regarding the activity of complexes II + III, no significant differences were found due to treatment with GSPE.

Concerning the last complex of the respiratory chain, complex IV or COX, not enough differences were found due to CAF or GSPE treatment. In this case, the activity of the complex was constant in all conditions and at all measured times. Finally, we also measured the activity of citrate synthase, which is considered a marker for mitochondrial activity. Despite the fact that there were no differences due to the CAF diet or GSPE treatment, there was a significant increase in the activity of this enzyme at ZT13 in rats treated diurnally with GSPE (ZT0), even exceeding the activity of the STD group (*p* = 0.001) (Supplementary Materials, Table S3). 

Altogether, these subtle results of mitochondrial complex activity suggest that there is a strong effect of the cafeteria diet at nearly all ZTs and that GSPE is able to restore them at discrete ZTs above all in the diurnal treatment. Additionally, it seems that the dynamics processes (fission and fusion) are more easily modulated, at certain ZTs, by GSPE than mitochondrial biogenesis.

### 3.6. GSPE Treatment Strongly Increases Concentrations of Metabolites of Tricarboxylic Acid Cycles in CAF-Fed Rats

**Figure 8:**
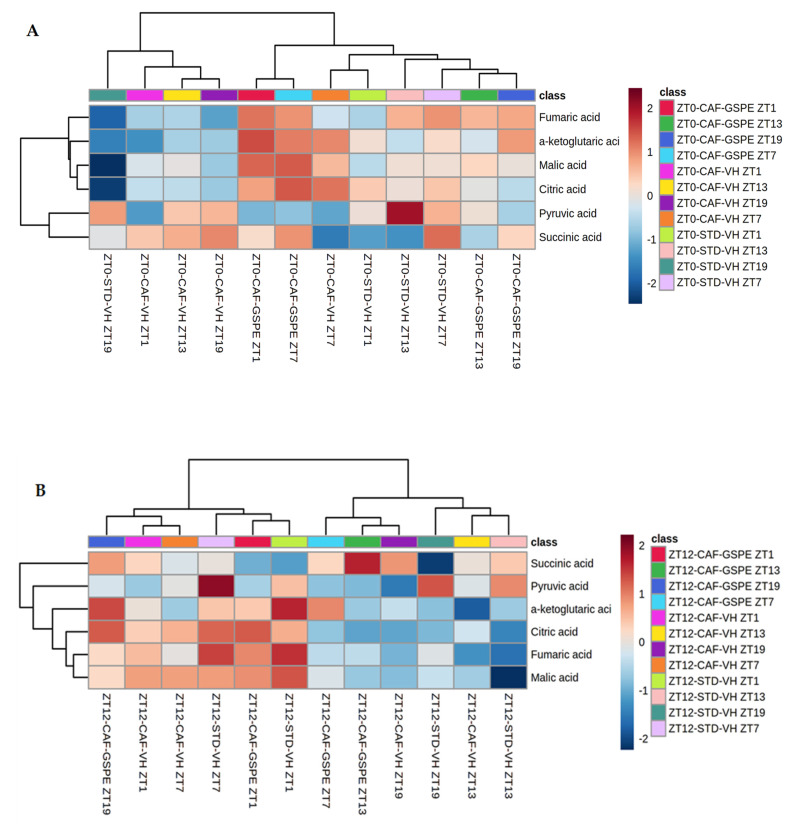
Heatmaps of TCA cycle metabolites in the liver. Rats were fed an STD or CAF diet and received a daily dosage of vehicle or GSPE in the morning (ZT0) (**A**) or at night (ZT12) (**B**). After 9 weeks, the rats were sacrificed at 9 a.m. (ZT1), 3 p.m. (ZT7), 9 p.m. (ZT13) or 3 a.m. (ZT19).

As a key regulator of metabolic pathways, mitochondria produce metabolites that are necessary for energy homeostasis, cell survival and proliferation. Therefore, six metabolites involved in one of the most important mitochondrial pathways, TCA cycle, were studied. When analyzing diurnal treatment (ZT0), Figure 8A shows that GSPE-treated animals exhibited a higher concentration of fumaric (*p* = 0.039), a-ketoglutaric (*p* = 0.013), malic (*p* = 0.047) and pyruvic acid (*p* = 0.024) metabolites at ZT1 than the STD and/or CAF-VH groups.

On the other hand, in the CAF-VH group at the same time point, a decrease in the concentration of these metabolites was observed. At ZT7 in the CAF-GSPE group, the concentration of the metabolites mentioned above remained high, whereas succinic acid levels began to rise (*p* = 0.047). On the other hand, 13 h after dosage, at ZT13, GSPE-treated animals displayed an important decrease in the concentration of metabolites, which continued in the same way at ZT19. CAF-VH animals showed lower concentrations of pyruvic acid than STD-VH animals at ZT13 (*p* = 0.040), as well as lower concentration of a-ketoglutaric acid than CAF-GSPE animals at ZT19 (*p* = 0.046). The lowest concentration was seen in STD animals at ZT19, where some metabolites values were two-fold lower than in CAF-GSPE animals (Supplementary Materials, Table S4.1).

This could mean that the cafeteria treatment depletes TCA metabolites due to the rich ATP environment arresting TCA and re-orienting these metabolites through anabolic purposes, i.e., lipid synthesis. On the other hand, GSPE could be partially restore this metabolic pathway. This is in agreement with the fact that the major restoration respiratory complexes induced by GSPE were also seen at several ZTs but always in the morning treatment. 

Regarding nocturnal treatment (ZT12), fumaric, a-ketoglutaric, malic and citric acid metabolites exhibit a higher concentration from ZT1 to ZT7 in VH-, CAF- and STD-diet animals; whereas in the case of GSPE-treated rats, higher amounts of these compounds were seen from ZT19 to ZT1, 6 h earlier than in the vehicles. What is more, lower concentrations of the aforementioned metabolites were seen from ZT13 to ZT19 in CAF-VH and STD-VH groups, while for CAF-GSPE animals, the lowest amount of these compounds was observed from ZT7 to ZT13 (Figure 8B). Succinic acid was significantly increased at ZT13 when treated with GSPE (*p* = 0.005), whereas pyruvic acid exhibited a significant decrease (*p* = 0.008) compared to CAF and STD VH, respectively (Supplementary Materials, Table S4.2). In this case, the fact that the rats were active in the nocturnal phase could explain the indistinctive results among treatments due to the fact that in this period, the ATP requirements increased, promoting TCA activity in all experimental groups. Nevertheless, we must also take into account that cafeteria treatment decreased complex activity. 

## 4. Discussion

It is well known that polyphenols can mitigate the detrimental effects of a CAF diet by decreasing body weight, blood pressure and blood glucose, as well as by improving lipid metabolism [31]. In this sense, our results show that GSPE is able to decrease body-weight gain and improve blood glucose levels of obese rats.

Furthermore, it has been previously shown that a dose of 25 mg GSPE/kg body weight per day administered for 30 days improved the homeostasis model assessment-insulin resistance index (HOMA-index). This was accompanied by downregulation of Pparg2, Glut4 and Irs1 in mesenteric white adipose tissue. Similarly, a chronic GSPE treatment of insulin-resistant 3T3-L1 adipocytes downregulated the mRNA levels of those adipocyte markers, although cells were still able to respond to the acute stimulation of glucose uptake. This indicates that the 25 mg/kg body weight per day GSPE treatment has a positive long-term effect on glucose homeostasis [48]. 

On the other hand, a limitation of our study is the number of animals used per group, although similar experiments on circadian rhythms [54,55,56,57] also used a low number of animals (around four) for each experimental time point.

Several lines of evidence suggest that the consumption of a high-fat diet causes misalignment of the liver molecular clock [58]. In this regard, our results indicate that a CAF diet causes a disruption in hepatic circadian-clock genes, as acrophases are shifted and rhythmicity of some core-clock genes are lost. Ribas-Latre and collaborators [59] studied the effect of GSPE on the clock system in HepG2 cells. According to their study, *Bmal1* was found to be the most GSPE-sensitive gene, since GSPE treatment strongly increased *Bmal1* expression. It is noteworthy that GSPE was also found to enhance the transcriptional activity of *Rorα*, which is an activator of *Bmal1* expression, suggesting that it might be responsible for the modulation of *Bmal1* by GSPE. This is in agreement with our results, as GSPE treatment is capable of restoring hepatic circadian misalignment of *Rorα* in both diurnal and nocturnal treatments, as well as of *Bmal1*, *Cry1* and *Per2* in animals treated at night (ZT12). Interestingly, Ribas-Latre et. al. [59] conducted another study where they administered GSPE in the morning (ZT0) or at night (ZT12) to healthy rats. 

When analyzing the expression of clock genes in the liver, they observed that the effect of GSPE on *Bmal1* only occurred in night-treated animals (ZT12) [60]. Hence, they postulated that GSPE could act as an adaptive element in the liver, enhancing the energy profile of rats and improving mitochondrial function and oxidation at night, during the active phase, as rats are nocturnal animals. Interestingly, we also found a major corrective effect on the misalignment of the circadian rhythm induced by a cafeteria diet in the nocturnal treatment (ZT12) in comparison to the diurnal treatment (ZT0). Additionally, this correlates with the increase in parameters suggestive of a fussed morphology at night. In this sense, it was observed that the morphology of the mitochondrial network shows circadian rhythmicity in cultured fibroblast, shifting from a tubular mitochondrial network at 16 h after synchronization to a greatly fragmented network 28 h after, coinciding with the rhythmicity of ATP content and oxidative phosphorylation. Moreover, *Per1*/*Per2* mutant mice lack these rhythms, and therefore, ATP levels are not cyclic, and the mitochondrial network remains fragmented at all time points [24]. Likewise, liver-specific *Bmal1* mutant mice exhibit swollen mitochondria, parallel with reduced levels of mitophagy proteins *Fis1* and *Pink1*, in addition to the inability to adapt to different nutritional conditions [22]. Furthermore, as has already been exposed, levels of mitochondrial fusion proteins *Mfn1* and *Opa1* are reduced in *Bmal1* knockout mice [4].

On the other hand, the recovery of mitochondrial complexes at ZT13 of diurnal GSPE administration are in agreement with changes in mitochondrial morphology (*Mfn1*, *Mfn2* and *Fis1*). In this regard, it has been shown that structural remodeling can act as a compensatory mechanism for inefficient mitochondrial ATP synthesis [61]. 

NAFLD is considered the hepatic manifestation of metabolic syndrome [62], where oxidative stress is the main mechanism of liver injury, leading to hepatic inflammation and fibrosis. As the major contributor to oxidative stress, mitochondrial dysfunction plays a significant role in this pathogenesis [63]. Thus, improper functioning of mitochondria could lead to the development of metabolic diseases, such as obesity, type 2 diabetes mellitus and NAFLD. Moreover, a large number of liver diseases are characterized by mitochondrial alterations [52]. Our results demonstrate that a CAF diet promotes a significant alteration in hepatic mitochondrial function, with three of the electron-transport-chain (ETC) components (complex I, II and III) exhibiting a decrease in activity. The ETC is the main site of ATP generation; therefore, we demonstrated that mitochondrial respiratory activity is hugely altered in rats consuming a CAF diet. Moreover, our results suggest that GSPE treatment could reduce, at least partially, the negative effect of the CAF diet over mitochondrial function. This last finding is in agreement with previously published studies about the beneficial effect of chronic supplementation with proanthocyanidins over mitochondrial activity and functionality in brown adipose tissue and skeletal muscle of obese rats [35,36].

To maintain healthy mitochondria, mitochondria are continuously formed and removed through dynamic processes called mitochondrial fission, fusion, mitophagy and biogenesis [64].Our results showed a differential effect on expression of fusion genes *Mfn1* and *Mfn2*, depending on the time of treatment administration. While in the morning treatment (ZT0), CAF was able to increase the expression of these genes and GSPE maintained similar expression levels as in healthy rats, in nocturnal treatment (ZT12), an increase in fusion gene expression was observed in GSPE-treated animals at ZT1. An excess of caloric intake causes an increase in glucose levels, which triggers the production of ROS, thereby generating mitochondrial oxidative stress [16]. It has been previously shown that mitochondrial oxidative stress causes mitochondrial fragmentation via differential modulation of mitochondrial fission–fusion proteins. On one side, some authors suggest overexpression of *Mfn1* and/or *Mfn2* causes mitochondrial dysfunction and cell death [65]. However, other authors demonstrate that overexpression of *Mfn2* improves liver fibrosis in hepatic stellate cells of mice [66]. In this regard, our results suggest that GSPE could enhance fusion expression when administrated at night (ZT12) to alleviate stress caused by a CAF-diet, thereby combining contents of partially damaged mitochondria as a form of complementation. Fission is necessary to create new mitochondria but also contributes to quality control by enabling the deletion of damaged mitochondria and could promote apoptosis during high levels of cellular stress. However, disruption of fission machinery is implicated in the development of neurodegenerative and metabolic diseases [67]. Our results show that the expression levels of fission genes in GSPE-treated rats were similar to those of STD-diet rats, whereas CAF-VH rats exhibited an increase in expression of these genes at most death time points. These findings are in accordance with several studies in which high-fat-diet-induced obesity mice showed overexpression of mitochondrial fission genes *Drp1* and *Fis1*, whereas inhibition or disruption of these genes led to an improvement in insulin resistance and mitochondrial dysfunction in skeletal muscle [7]. Furthermore, a decrease in mitochondrial fission has also been found to ameliorate hepatic steatosis and to protect the liver against metabolic deterioration in a murine model of NAFLD [68,69]. 

Food-derived fatty acids, carbohydrates and proteins are oxidized inside mitochondria via β-oxidation and TCA cycle [63]. Thereby, TCA cycle, also known as Krebs cycle, plays an important role as an energy supply for metabolism. Moreover, it produces important intermediates involved in gluconeogenesis, lipolysis and neurotransmitter synthesis, among others. It is also known that TCA cycle and the ETC are intimately related, since byproduct generation of 3 nicotinamide adenine dinucleotide (NADH) and 1 flavin adenine dinucleotide (FADH2) by TCA cycle feeds ETC complex I (NADH dehydrogenase) and complex II succinate dehydrogenase (SDH), respectively. Furthermore, oxidation of the aforementioned byproducts in complexes I and II is necessary to maintain the proper functioning of the TCA cycle [70]. For these reasons, we evaluated the concentration of the TCA cycle metabolites in the liver and observed higher concentrations of these metabolites in morning GSPE-treated animals (ZT0). It is possible that a correlation exists between this result and the tendency to increase complex I activity when GSPE was supplied in the morning at ZT0. Moreover, not only are concentrations of these metabolites strongly deceased in CAF-fed animals in both diurnal and nocturnal treatments, but mitochondrial respiratory activity is also hugely altered. This could be partially attributed to the accumulation of NADH due to malfunctioning of ETC. The regulatory enzymes of the TCA cycle are inhibited by NADH. Therefore, we suggest that the TCA cycle turns off in CAF-fed animals as a consequence of ETC dysfunction. Interestingly, we observed that as expression of *Fis1* and/or *Drp1* in CAF-VH group decreases, concentrations of TCA metabolites increase at the same time of death. In the same way, an enhancement of fission gene expression in CAF-VH animals correlates with a decrease in TCA metabolite concentrations. Considering the significant role of mitochondrial dynamics and respiratory-chain activity in mitochondrial and cellular functions, it could be suggested that obesity and excessive caloric intake cause an imbalance of these processes, leading to mitochondrial dysfunction and contributing to the development of hepatic metabolic impairment.

Several studies go along with the idea that maintaining circadian rhythm is essential, as it plays a pivotal role in hepatic metabolism and mitochondrial function [20]. In this sense, Kim and collaborators [71] demonstrated liver-specific inhibition of *Rorα* induced abnormal mitochondrial function, thus worsening NAFLD in mice with a high-fat diet. They evidenced *Rorα* that increased the rate of oxygen consumption and the expression of genes involved in mitochondrial quality control [71]. Moreover, Jacobi and colleges revealed circadian control of *Bmal1* over expression of mitochondrial-dynamics-related genes. Their study exposed that the hepatic deletion of *Bmal1* causes oxidative stress and damages mitochondria [22]. In this sense, our results show that circadian oscillations of *Rorα* and *Bmal1* were disrupted in the CAF-diet group and that GSPE was able to restore this disruption of both clock genes when treated at night (ZT12). Therefore, we can suggest that chronic consumption of GSPE could be useful to ameliorate obesity-induced mitochondrial dysfunction by improving the disturbance of circadian rhythm. 

Finally, in this experiment, GSPE was administered at 25 mg of GSPE/ kg of body weight. This dose, using a translation of animal to human doses [72] and estimating the daily intake for a 70 kg human, corresponds to an intake of 284 mg of GSPE/day. This GSPE intake can be achieved in humans with a polyphenol-rich diet. For example, in Spanish adults, the mean dietary flavonoid intake is 313.26 mg/day, with proanthocyanidins comprising 60.1% [73]. Although experimental data obtained in rats cannot be directly extrapolated to humans, the fact that GSPE is able to restore the rhythm of clock hepatic genes (given this correction more evident on the nocturnal treatment), as well as other mitochondrial parameters and TCA-related metabolites, suggests that the inclusion of proanthocyanidin-rich foods in the diets of obese humans could be a good strategy to improve their hepatic circadian and mitochondrial alterations.

## 5. Conclusions

In summary, dietary supplementation with GSPE caused a decrease in body-weight gain in animals fed a CAF diet. Moreover, other significant metabolic conditions associated with metabolic syndrome were also improved after GSPE administration. In particular, an improvement in serum glucose levels was observed compared to CAF non-treated animals. Furthermore, GSPE was able to restore the rhythmicity of some core-clock genes in the liver that were totally disrupted by a CAF diet. In addition, our results demonstrate significant alteration in hepatic mitochondrial function caused by an obesogenic diet intake, such as a decrease in mitochondrial complex I, II and II + III activities; an increase in the expression of mitochondrial fission genes, which can result in fragmented mitochondria that are susceptible to apoptosis; and a strong decease in the concentrations of TCA cycle metabolites. In this sense, our results suggest that GSPE treatment could ameliorate the effect of the CAF diet over mitochondrial function. Notably, the major improvements in GSPE supplementations were seen at the ZT12 dosage. This may be partially explained by the fact that GSPE enhances the energy profile of rats and improves mitochondrial function and oxidation in the liver at night, during the active phase when rats are metabolically more active, as rats are nocturnal animals. Nevertheless, further studies are needed to elucidate the molecular mechanisms involved in these events. 

## Figures and Tables

**Table 1:** Nucleotide sequences of primers used for real-time quantitative PCR.

Gene	Forward Primer (5′ to 3′)	Reverse Primer (5′ to 3′)
*Bmal1*	GTAGATCAGAGGGCGACGGCTA	CTTGTCTGTAAAACTTGCCTGTGAC
*Cry1*	TGGAAGGTATGCGTGTCCTC	TCCAGGAGAACCTCCTCACG
*Drp1*	CCAGGAATGACCAAGGTCCC	CCTCGTCCATCAGGTCCAAC
*Fis1*	GCACGCAGTTTGAATACGCC	CTGCTCCTCTTTGCTACCTTTGG
*Mfn1*	CCTTGTACATCGATTCCTGGGTTC	CCTGGGCTGCATTATCTGGTG
*Mfn2*	GATGTCACCACGGAGCTGGA	AGAGACGCTCACTCACTTTG
*Nampt*	CTCTTCACAAGAGACTGCCG	TTCATGGTCTTTCCCCCACG
*Nr1d1*	ACAGCTGACACCACCCAGATC	CATGGGCATAGGTGAAGATTTCT
*Pcg1α*	AGAGTCACCAAATGACCCCAAG	TTGGCTTTATGAGGAGGAGTCG
*Per2*	CGGACCTGGCTTCAGTTCAT	AGGATCCAAGAACGGCACAG
*Ppia*	CCAAACACAAATGGTTCCCAGT	ATTCCTGGACCCAAAACGCT
*Rorα*	CCCGATGTCTTCAAATCCTTAGG	TCAGTCAGATGCATAGAACACAAACTC

**Table 2:** Biochemical parameters of rats fed with STD or CAF diet supplemented with vehicle or GSPE.

			Glucose (mg/dL)	Cholesterol (mg/dL)	Triglycerides (mg/dL)	NEFA (mg/dL)
ZT0	ZT1	STD-VH	90.11 ± 2.68	90.96 ± 13.33	64.07 ± 5.81	21.93 ± 3.84
		CAF-VH	96.22 ± 5.25	95.99 ± 7.07	108.5 ± 6.17 **	24.71 ± 2.46
		CAF-GSPE	101.72 ± 7.7	100.55 ± 8.53	107.41 ± 19.36	21.12 ± 1.23
	ZT7	STD-VH	89.45 ± 3.38	101.17 ± 4.2	118.11 ± 14.71	32.4 ± 2.23
		CAF-VH	110.84 ± 4.22 **	134.24 ± 14.32 *	319.19 ± 15.87 ***	31.15 ± 2.2
		CAF-GSPE	114.6 ± 6.01	142.02 ± 13.78	280.27 ± 42.88	33.17 ± 1.51
	ZT13	STD-VH	89.6 ± 3.67	87.6 ± 6.68	53.13 ± 9.52	25.38 ± 6.2
		CAF-VH	111.29 ± 6 *	112.86 ± 22.76	204.94 ± 57.47 *	32.62 ± 3.96
		CAF-GSPE	96.13 ± 3.89 #	79.6 ± 7.44	136.11 ± 32.03	35.5 ± 1.05
	ZT19	STD-VH	79.62 ± 4.25	85.75 ± 9.53	50.22 ± 6.36	22.23 ± 3.02
		CAF-VH	96.2 ± 6.8	97.05 ± 4.44	133.68 ± 18 **	28.66 ± 2.04
		CAF-GSPE	97.38 ± 11.51	82.53 ± 9.56	118.93 ± 11.97	31.92 ± 2.97
ZT12	ZT1	STD-VH	85.7 ± 4.92	119.64 ± 17.95	84.23 ± 19.56	31.46 ± 10.85
		CAF-VH	103.82 ± 4.64 *	88.39 ± 10.1	122.92 ± 10.74	26.58 ± 2.58
		CAF-GSPE	108.95 ± 8.27	92.68 ± 7.49	181.82 ± 27.14	30.66 ± 7.37
	ZT7	STD-VH	87.28 ± 8.97	134.24 ± 14.51	163.51 ± 19.99	30.14 ± 2.47
		CAF-VH	116.33 ± 6.05 *	122.37 ± 17.35	320.27 ± 48.57 *	32.74 ± 2.73
		CAF-GSPE	125.87 ± 7.78	151.36 ± 13.35	366.76 ± 45.42	34.47 ± 3.99
	ZT13	STD-VH	85.49 ± 4.15	100.51 ± 14.49	49.84 ± 3.83	27.29 ± 2.74
		CAF-VH	124.13 ± 10.98 *	119.27 ± 13.91	189.03 ± 39.51 *	33.28 ± 4.73
		CAF-GSPE	112.93 ± 9.55	118.23 ± 17.02	183.64 ± 55.82	34.24 ± 3.52
	ZT19	STD-VH	81.01 ± 7.4	93.55 ± 6.9	65.25 ± 6.78	31.02 ± 2.14
		CAF-VH	125.05 ± 10.89 *	134.03 ± 19.23 *	213.53 ± 51.16 *	30.18 ± 2.07
		CAF-GSPE	106.35 ± 14.1	128.44 ± 30.01	179.87 ± 44.01	32.26 ± 8.15

Serum parameters of rats fed with standard diet (STD) or cafeteria diet (CAF) for 9 weeks and supplemented with vehicle or grape-seed proanthocyanidin extract (GSPE) for the last 4 weeks. Values are expressed as the mean ± standard error of the mean (SEM) (*n* = 4). * The effect of diet within vehicle groups (Student’s *t* test, * *p* ≤ 0.05, ** *p* ≤ 0.01, *** *p* ≤ 0.001); # indicates tendency between cafeteria-diet–vehicle (CAF-VH) and cafeteria-diet–grape-seed proanthocyanidin extract (CAF-GSPE) using Student’s *t* test (*p* = 0.1–0.051).

## Data Availability

The data presented in this study are available on request from the corresponding author. The data are not publicly available due to lack of platform to publish them.

## Supplementary Materials

The following are available online at https://www.mdpi.com/article/10.3390/nu14040774/s1, Table S1: Circadian parameters of liver clock genes, Table S2: Comparison of circadian parameters of liver clock genes between groups, Table S3: Measurement of ETC components in the liver, Table S4.1: Metabolites of the TCA cycle in the liver at ZT0, Table S4.2: Metabolites of the TCA cycle in the liver at ZT12.
