# Supplementary material for: Time-of-Day Circadian Modulation of Grape-Seed Procyanidin Extract (GSPE) in Hepatic Mitochondrial Dynamics in Cafeteria-Diet-Induced Obese Rats
Source: Nutrients. 2022 Feb 12;14(4):774. doi: 10.3390/nu14040774 (PMC8876123; doi:10.3390/nu14040774)
Supplement: Supplementary file 1 [file nutrients-14-00774-s001.zip › nutrients-1549434-supplementary.pdf]

Table S1. Circadian parameters of liver clock genes.

			p	amplitude	*p*(amplitude)	acrophase (h)	*p*(acrophase)	MESOR
Bmal1	ZT0	STD-VH	**0.000**	0.488	**0.000**	0.566	**0.011**	0.478
		CAF-VH	**0.000**	0.579	**0.000**	0.187	**0.000**	0.553
		CAF-GSPE	**0.000**	0.591	**0.000**	0.59	0.518	0.59
	ZT12	STD-VH	**0.000**	0.479	**0.000**	22.638	**0.007**	0.394
		CAF-VH	**0.000**	0.446	**0.000**	1.51	0.111	0.477
		CAF-GSPE	**0.001**	0.369	**0.000**	23.055	**0.000**	0.326
Cry1	ZT0	STD-VH	**0.001**	0.503	**0.000**	20.629	**0.000**	0.742
		CAF-VH	**0.000**	0.615	**0.000**	21.272	**0.000**	0.816
		CAF-GSPE	**0.000**	0.672	**0.000**	21.1	**0.000**	0.856
	ZT12	STD-VH	**0.001**	0.753	**0.000**	18.664	**0.000**	0.969
		CAF-VH	**0.000**	0.497	**0.000**	21.09	**0.000**	0.705
		CAF-GSPE	**0.000**	0.753	**0.000**	18.577	**0.000**	0.71
Per2	ZT0	STD-VH	**0.003**	0.807	**0.000**	16.58	**0.000**	1.295
		CAF-VH	**0.000**	1.205	**0.000**	16.017	**0.000**	1.762
		CAF-GSPE	**0.000**	1.293	**0.000**	16.58	**0.000**	1.651
	ZT12	STD-VH	**0.000**	1.407	**0.000**	16.304	**0.000**	2.067
		CAF-VH	**0.000**	2.264	**0.000**	13.519	**0.000**	3.035
		CAF-GSPE	**0.000**	1.408	**0.000**	15.271	**0.000**	1.719
Nr1d1	ZT0	STD-VH	**0.004**	22.855	**0.000**	11.207	**0.000**	16.15
		CAF-VH	**0.000**	34.868	**0.000**	9.386	**0.000**	25.125
		CAF-GSPE	**0.000**	30.889	**0.000**	9.692	**0.000**	22.919
	ZT12	STD-VH	**0.000**	36.283	**0.000**	8.038	**0.000**	23.206
		CAF-VH	**0.001**	34.974	**0.000**	8.312	**0.000**	23.648
		CAF-GSPE	**0.000**	23.224	**0.000**	10.424	**0.000**	17.181
Ror-α	ZT0	STD-VH	0.184	0.174	**0.049**	20.905	**0.000**	0.921
		CAF-VH	**0.000**	0.376	**0.000**	16.541	**0.000**	0.961
		CAF-GSPE	**0.000**	0.42	**0.000**	21.71	**0.000**	1.078
	ZT12	STD-VH	**0.020**	0.261	**0.001**	19.361	**0.000**	0.56
		CAF-VH	0.307	0.128	0.107	7.621	**0.001**	0.623
		CAF-GSPE	**0.046**	0.225	**0.005**	21.231	**0.000**	0.431
Nampt	ZT0	STD-VH	**0.001**	2.523	**0.000**	14.497	**0.000**	2.495
		CAF-VH	**0.003**	3.915	**0.000**	14.361	**0.000**	4.3
		CAF-GSPE	**0.031**	2.493	**0.002**	16.522	**0.000**	3.437
	ZT12	STD-VH	0.062	0.624	**0.008**	17.284	**0.000**	1.26
		CAF-VH	**0.003**	1.295	**0.000**	13.401	**0.000**	1.576
		CAF-GSPE	**0.001**	1.224	**0.000**	14.488	**0.000**	1.26

Rats fed with standard diet (STD) or cafeteria diet (CAF) were daily dosage with vehicle or grape seed procyanidins extract (GSPE) in the morning (ZT0) or at night (ZT12) and after 9 weeks were sacrificed at 9 a.m. (ZT1), 3 p.m. (ZT7), 9 p.m. (ZT13) or 3 a.m. (ZT19). Acrophase is the time at which the peak of a rhythm occurs (h); Amplitude is the difference between the peak and the mean value of a wave; MESOR is a circadian rhythm-adjusted mean. The values are the estimation of circadian parameters obtained by Cosinor method. The statistically significant p-values ($p < 0.05$) are highlighted in bold.

Table S2. Comparison of circadian parameters of liver clock genes between groups.

			p	d_amplitude	*p*(d_amplitude)	d_acrophase	*p*(d_acrophase)
Bmal1	ZT0	STD-VH vs. CAF-VH	**0.000**	0.091	0.149	0.099	0.406
		CAF-VH vs. CAF-GSPE	**0.000**	0.012	0.845	−0.106	0.278
	ZT12	STD-VH vs. CAF-VH	**0.000**	−0.033	0.689	5.531	**0.000**
		CAF-VH vs. CAF-GSPE	**0.000**	−0.077	0.419	−5.64	**0.000**
Cry1	ZT0	STD-VH vs. CAF-VH	**0.000**	0.113	0.369	−0.168	0.459
		CAF-VH vs. CAF-GSPE	**0.000**	0.057	0.598	0.045	0.793
	ZT12	STD-VH vs. CAF-VH	**0.000**	−0.256	0.149	−0.635	**0.036**
		CAF-VH vs. CAF-GSPE	**0.000**	0.09	0.424	0.658	**0.001**
Per2	ZT0	STD-VH vs. CAF-VH	**0.000**	0.398	0.055	0.148	0.499
		CAF-VH vs. CAF-GSPE	**0.000**	0.089	0.615	−0.148	0.301
	ZT12	STD-VH vs. CAF-VH	**0.000**	0.857	**0.027**	0.729	**0.001**
		CAF-VH vs. CAF-GSPE	**0.000**	−0.856	**0.048**	−0.458	0.071
Nr1d1	ZT0	STD-VH vs. CAF-VH	**0.000**	12.013	**0.047**	0.477	**0.033**
		CAF-VH vs. CAF-GSPE	**0.000**	−3.979	0.308	−0.08	0.506
	ZT12	STD-VH vs. CAF-VH	**0.000**	−1.308	0.881	−0.072	0.763
		CAF-VH vs. CAF-GSPE	**0.000**	−11.75	0.122	−0.553	**0.040**
Ror-α	ZT0	STD-VH vs. CAF-VH	**0.001**	0.203	0.068	1.143	**0.022**
		CAF-VH vs. CAF-GSPE	**0.000**	0.044	0.662	−1.353	**0.000**
	ZT12	STD-VH vs. CAF-VH	**0.037**	−0.133	0.235	3.074	**0.000**
		CAF-VH vs. CAF-GSPE	**0.024**	0.097	0.387	−3.563	**0.000**
Nampt	ZT0	STD-VH vs. CAF-VH	**0.000**	1.392	0.181	0.036	0.918
		CAF-VH vs. CAF-GSPE	**0.001**	−1.422	0.248	−0.566	0.180
	ZT12	STD-VH vs. CAF-VH	**0.001**	0.671	0.077	1.017	**0.033**
		CAF-VH vs. CAF-GSPE	**0.000**	−0.071	0.852	−0.285	0.345

Rats fed with STD or CAF were daily dosage with vehicle or GSPE in the morning (ZT0) or at night (ZT12) and after 9 weeks were sacrificed at 9 a.m. (ZT1), 3 p.m. (ZT7), 9 p.m. (ZT13) or 3 a.m. (ZT19). Acrophase is the time at which the peak of a rhythm occurs ([h], hours); Amplitude is the difference between the peak and the mean value of a wave; MESOR is a circadian rhythm-adjusted mean. The first column (*p*) indicates if there are significant differences between two circadian rhythms. The circadian parameter values are the differences between two groups of each circadian parameter. The statistically significant p-values ($p < 0.05$) are highlighted in bold.

Table S3. Measurement of ETC components in the liver.

			Citrate synthase mU/mg protein	Complex I mU/mg protein	Complex II mU/mg protein	Complex II + II mU/mg protein	COX mU/mg protein
ZT0	ZT1	STD-VH	62.55 ± 6.08	33.95 ± 5.68	118.40 ± 15.08	8.74 ± 1.67	71.69 ± 6.08
		CAF-VH	64.81 ± 6.52	25.54 ± 6.79	80.07 ± 5.57 **	4.55 ± 0.66 **	66.92 ± 10.38
		CAF-GSPE	56.91 ± 3.04	34.86 ± 17.53	74.46 ± 15.99	3.86 ± 1.25	62.51 ± 24.41
	ZT7	STD-VH	60.23 ± 9.33	25.62 ± 1.85	99.40 ± 19.49	6.53 ± 1.86	69.85 ± 18.45
		CAF-VH	60.05 ± 6.28	15.52 ± 2.66 ***	63.28 ± 4.12 **	4.02 ± 0.58 *	66.76 ± 11.18
		CAF-GSPE	59.88 ± 8.09	23.38 ± 3.59 $	73.66 ± 19.14	4.59 ± 1.34	67.68 ± 15.91
	ZT13	STD-VH	70.28 ± 9.0	33.24 ± 16.81	122.42 ± 25.57	7.22 ± 2.58	58.71 ± 12.70
		CAF-VH	64.31 ± 2.44	20.33 ± 4.51	69.25 ± 17.96 *	3.98 ± 0.94 #	54.83 ± 5.00
		CAF-GSPE	74.67 ± 2.57 $$	32.33 ± 4.71 $	112.02 ± 12.16 $$	60.8 ± 1.99 +	56.46 ± 4.48
	ZT19	STD-VH	69.49 ± 7.01	37.74 ± 3.34	144.27 ± 11.08	8.82 ± 1.64	58.21 ± 11.96
		CAF-VH	72.49 ± 4.64	32.53 ± 4.06 $	108.75 ± 16.10 **	5.24 ± 0.70 **	51.22 ± 11.64
		CAF-GSPE	70.23 ± 8.78	22.88 ± 3.00 $$	86.97 ± 8.35 +	4.61 ± 0.75	57.59 ± 11.44
ZT12	ZT1	STD-VH	65.02 ± 7.97	28.71 ± 12.08	100.19 ± 19.64	6.41 ± 1.28	65.95 ± 9.85
		CAF-VH	60.97 ± 4.69	26.26 ± 6.81	78.31 ± 13.99	4.75 ± 1.06 #	57.39 ± 12.80
		CAF-GSPE	63.93 ± 6.21	26.41 ± 5.10	79.91 ± 19.67	5.33 ± 3.11	54.90 ± 4.42
	ZT7	STD-VH	59.72 ± 4.06	41.60 ± 7.40	112.19 ± 17.75	7.25 ± 1.29	56.98 ± 10.80
		CAF-VH	59.32 ± 3.77	23.12 ± 7.33 *	63.43 ± 9.66 **	4.28 ± 0.94 **	54.85 ± 6.49
		CAF-GSPE	57.36 ± 2.32	31.85 ± 12.93	78.61 ± 9.95 +	4.53 ± 1.07	57.65 ± 2.35
	ZT13	STD-VH	68.75 ± 8.66	33.91 ± 6.82	126.38 ±12.90	8.46 ± 2.03	56.22 ± 10.17
		CAF-VH	64.66 ± 5.09	22.63 ± 4.91 *	84.67 ± 7.61 ***	4.94 ± 0.70 *	47.79 ± 6.68
		CAF-GSPE	58.93 ± 3.03	23.86 ± 6.35	72.84 ± 11.18	4.02 ± 0.86	43.98 ± 5.64
	ZT19	STD-VH	69.00 ± 5.11	42.16 ± 17.59	132.48 ± 26.19	8.59 ± 4.10	56.59 ± 11.18
		CAF-VH	63.16 ± 6.68	22.95 ± 12.47	69.47 ± 40.41 *	3.49 ±2.50 +	55.52 ± 19.91
		CAF-GSPE	61.66 ± 8.12	23.06 ± 8.14	84.00 ±18.94	4.45 ± 2.16	45.36 ± 11.36

Rats fed with STD or CAF were daily dosage with vehicle or GSPE in the morning (ZT0) or at night (ZT12) and after 9 weeks were sacrificed at 9 a.m. (ZT1), 3 p.m. (ZT7), 9 p.m. (ZT13) or 3 a.m. (ZT19). The values are the mean ± standard error of the mean (SEM). ($n = 4$). * The effect of diet within vehicle groups (Student's t test, $p < 0.05$); $ The effect of GSPE consumption within CAF groups (Student's t test, $p < 0.05$); + Indicates tendency between CAF-VH and CAF-GSPE using Student´s t test ($p = 0.1–0.051$). COX Cytochrome c oxidase.

Table S4.1 Metabolites of the TCA cycle in the liver.

ZT	Metabolite	ZT0-STD-VH vs. ZT0-CAF-VH				ZT0-CAF-VH vs. ZT0-CAF-GSPE				ZT0-STD-VH vs. ZT0-CAF-GSPE			
		**ZT0-STD-VH**	**ZT0-CAF-VH**	***p*-value**	**FC**	**ZT0-CAF-VH**	**ZT0-CAF-GSPE**	***p*-value**	**FC**	**ZT0-STD-VH**	**ZT0-CAF-GSPE**	***p*-value**	**FC**
ZT1	Pyruvic acid	0.46 ± 0.03	0.32 ± 0.06	0.068	0.7	0.32 ± 0.06	0.34 ± 0.02	0.816	1.1	0.46 ± 0.03	0.34 ± 0.02	**0.024**	0.7
	Succinic acid	0.75 ± 0.08	1.1 ± 0.2	0.155	1.5	1.1 ± 0.2	1.06 ± 0.25	0.886	1	0.75 ± 0.08	1.06 ± 0.25	0.242	1.4
	Fumaric acid	6.37 ± 0.69	6.54 ± 1.13	0.905	1	6.54 ± 1.13	9.41 ± 0.87	0.118	1.4	6.37 ± 0.69	9.41 ± 0.87	**0.039**	1.5
	Malic acid	2.61 ± 0.37	2.86 ± 0.54	0.709	1.1	2.86 ± 0.54	3.79 ± 0.11	0.212	1.3	2.61 ± 0.37	3.79 ± 0.11	**0.047**	1.5
	α-ketoglutaric acid	0.04 ± 0.01	0.02 ± 0	0.150	0.5	0.02 ± 0	0.05 ± 0.01	**0.013**	2.5	0.04 ± 0.01	0.05 ± 0.01	0.245	1.3
	Citric acid	0.37 ± 0.08	0.26 ± 0.07	0.332	0.7	0.26 ± 0.07	0.43 ± 0.06	0.128	1.7	0.37 ± 0.08	0.43 ± 0.06	0.600	1.2
ZT7	Pyruvic acid	0.56 ± 0.06	0.32 ± 0.01	**0.005**	0.6	0.32 ± 0.01	0.36 ± 0.03	0.349	1.1	0.56 ± 0.06	0.36 ± 0.03	**0.019**	0.6
	Succinic acid	1.27 ± 0.15	0.73 ± 0.14	**0.038**	0.6	0.73 ± 0.14	1.18 ± 0.12	**0.047**	1.6	1.27 ± 0.15	1.18 ± 0.12	0.658	0.9
	Fumaric acid	9.17 ± 1.48	7.15 ± 1.37	0.356	0.8	7.15 ± 1.37	8.98 ± 0.99	0.322	1.3	9.17 ± 1.48	8.98 ± 0.99	0.918	1
	Malic acid	3 ± 0.51	3.56 ± 0.84	0.593	1.2	3.56 ± 0.84	3.98 ± 0.56	0.688	1.1	3 ± 0.51	3.98 ± 0.56	0.242	1.3
	α-ketoglutaric acid	0.04 ± 0.01	0.05 ± 0.01	0.307	1.3	0.05 ± 0.01	0.05 ± 0.01	0.944	1	0.04 ± 0.01	0.05 ± 0.01	0.263	1.3
	Citric acid	0.42 ± 0.16	0.52 ± 0.04	0.586	1.2	0.52 ± 0.04	0.6 ± 0.12	0.508	1.2	0.42 ± 0.16	0.6 ± 0.12	0.392	1.4
ZT13	Pyruvic acid	0.86 ± 0.1	0.54 ± 0.07	**0.040**	0.6	0.54 ± 0.07	0.47 ± 0.03	0.376	0.9	0.86 ± 0.1	0.47 ± 0.03	**0.010**	0.5
	Succinic acid	0.73 ± 0.08	1.16 ± 0.19	0.083	1.6	1.16 ± 0.19	0.88 ± 0.16	0.311	0.8	0.73 ± 0.08	0.88 ± 0.16	0.436	1.2
	Fumaric acid	8.4 ± 0.73	6.38 ± 0.61	0.079	0.8	6.38 ± 0.61	8.4 ± 0.86	0.104	1.3	8.4 ± 0.73	8.4 ± 0.86	0.999	1
	Malic acid	2.91 ± 0.27	2.81 ± 0.25	0.793	1	2.81 ± 0.25	3.11 ± 0.48	0.597	1.1	2.91 ± 0.27	3.11 ± 0.48	0.726	1.1
	α-ketoglutaric acid	0.03 ± 0.01	0.03 ± 0	0.674	1	0.03 ± 0	0.03 ± 0	0.495	1	0.03 ± 0.01	0.03 ± 0	0.883	1
	Citric acid	0.3 ± 0.05	0.22 ± 0.02	0.209	0.7	0.22 ± 0.02	0.29 ± 0.06	0.334	1.3	0.3 ± 0.05	0.29 ± 0.06	0.893	1
ZT19	Pyruvic acid	0.62 ± 0.11	0.55 ± 0.03	0.568	0.9	0.55 ± 0.03	0.39 ± 0.06	0.053	0.7	0.62 ± 0.11	0.39 ± 0.06	0.112	0.6
	Succinic acid	1.02 ± 0.21	1.23 ± 0.16	0.464	1.2	1.23 ± 0.16	1.08 ± 0.2	0.589	0.9	1.02 ± 0.21	1.08 ± 0.2	0.843	1.1
	Fumaric acid	4.74 ± 0.52	5.56 ± 0.63	0.352	1.2	5.56 ± 0.63	8.79 ± 1.26	0.061	1.6	4.74 ± 0.52	8.79 ± 1.26	**0.025**	1.9
	Malic acid	1.63 ± 0.18	2.43 ± 0.3	0.061	1.5	2.43 ± 0.3	3.02 ± 0.63	0.427	1.2	1.63 ± 0.18	3.02 ± 0.63	0.076	1.9
	α-ketoglutaric acid	0.02 ± 0	0.03 ± 0	0.083	1.5	0.03 ± 0	0.04 ± 0.01	**0.046**	1.3	0.02 ± 0	0.04 ± 0.01	**0.008**	2
	Citric acid	0.09 ± 0.02	0.2 ± 0.04	**0.042**	2.2	0.2 ± 0.04	0.23 ± 0.04	0.660	1.2	0.09 ± 0.02	0.23 ± 0.04	**0.022**	2.6

Metabolites of the tricarboxylic acid (TCA) cycle in the liver of rats fed with STD or CAF diet were daily dosage with vehicle or GSPE in the morning (ZT0) and after 9 weeks were sacrificed at 9 a.m. (ZT1), 3 p.m. (ZT7), 9 p.m. (ZT13) or 3 a.m. (ZT19). The statistically significant p-values ($p < 0.05$) are highlighted in bold.

Table S4.2 Metabolites of the TCA cycle in the liver.

ZT	Metabolite	ZT12-STD-VH vs. ZT12-CAF-VH				ZT12-CAF-VH vs. ZT12-CAF-GSPE				ZT12-STD-VH vs. ZT12-CAF-GSPE			
		**ZT12-STD-VH**	**ZT12-CAF-VH**	***p*-value**	**FC**	**ZT12-CAF-VH**	**ZT12-CAF-GSPE**	**p-value**	**FC**	**ZT12-STD-VH**	**ZT12-CAF-GSPE**	***p*-value**	**FC**
ZT1	Pyruvic acid	0.46 ± 0.04	0.37 ± 0.04	0.128	0.8	0.37 ± 0.04	0.39 ± 0.06	0.808	1.1	0.46 ± 0.04	0.39 ± 0.06	0.343	0.8
	Succinic acid	0.9 ± 0.24	1.13 ± 0.17	0.470	1.3	1.13 ± 0.17	0.98 ± 0.35	0.724	0.9	0.9 ± 0.24	0.98 ± 0.35	0.846	1.1
	Fumaric acid	13.34 ± 2.3	9.71 ± 0.81	0.186	0.7	9.71 ± 0.81	11.19 ± 1.74	0.468	1.2	13.34 ± 2.3	11.19 ± 1.74	0.484	0.8
	Malic acid	4.55 ± 0.66	3.76 ± 0.23	0.301	0.8	3.76 ± 0.23	3.99 ± 0.5	0.696	1.1	4.55 ± 0.66	3.99 ± 0.5	0.525	0.9
	α-ketoglutaric acid	0.04 ± 0.01	0.03 ± 0.01	0.482	0.8	0.03 ± 0.01	0.04 ± 0.01	0.804	1.3	0.04 ± 0.01	0.04 ± 0.01	0.650	1
	Citric acid	0.31 ± 0.06	0.32 ± 0.11	0.933	1	0.32 ± 0.11	0.41 ± 0.15	0.637	1.3	0.31 ± 0.06	0.41 ± 0.15	0.548	1.3
ZT7	Pyruvic acid	0.62 ± 0.07	0.42 ± 0.05	**0.045**	0.7	0.42 ± 0.05	0.36 ± 0.03	0.344	0.9	0.62 ± 0.07	0.36 ± 0.03	**0.012**	0.6
	Succinic acid	1.05 ± 0.14	1.02 ± 0.14	0.874	1	1.02 ± 0.14	1.13 ± 0.17	0.652	1.1	1.05 ± 0.14	1.13 ± 0.17	0.754	1.1
	Fumaric acid	12.51 ± 1.53	8.5 ± 1.14	0.081	0.7	8.5 ± 1.14	7.94 ± 1.71	0.796	0.9	12.51 ± 1.53	7.94 ± 1.71	0.094	0.6
	Malic acid	3.9 ± 0.55	3.81 ± 0.39	0.892	1	3.81 ± 0.39	3.01 ± 0.63	0.325	0.8	3.9 ± 0.55	3.01 ± 0.63	0.329	0.8
	α-ketoglutaric acid	0.03 ± 0	0.03 ± 0.01	0.568	1	0.03 ± 0.01	0.04 ± 0	0.355	1.3	0.03 ± 0	0.04 ± 0	0.667	1.3
	Citric acid	0.37 ± 0.1	0.3 ± 0.05	0.580	0.8	0.3 ± 0.05	0.22 ± 0.06	0.365	0.7	0.37 ± 0.1	0.22 ± 0.06	0.273	0.6
ZT13	Pyruvic acid	0.5 ± 0.03	0.41 ± 0.05	0.188	0.8	0.41 ± 0.05	0.35 ± 0.03	0.341	0.9	0.5 ± 0.03	0.35 ± 0.03	**0.008**	0.7
	Succinic acid	1.13 ± 0.09	1.04 ± 0.08	0.487	0.9	1.04 ± 0.08	1.45 ± 0.05	**0.005**	1.4	1.13 ± 0.09	1.45 ± 0.05	**0.021**	1.3
	Fumaric acid	5.55 ± 0.64	6.11 ± 0.67	0.570	1.1	6.11 ± 0.67	7.69 ± 0.98	0.233	1.3	5.55 ± 0.64	7.69 ± 0.98	0.118	1.4
	Malic acid	1.57 ± 0.22	2.51 ± 0.21	**0.023**	1.6	2.51 ± 0.21	2.48 ± 0.31	0.946	1	1.57 ± 0.22	2.48 ± 0.31	0.053	1.6
	α-ketoglutaric acid	0.03 ± 0	0.02 ± 0	0.315	0.7	0.02 ± 0	0.03 ± 0.01	0.356	1.5	0.03 ± 0	0.03 ± 0.01	0.942	1
	Citric acid	0.18 ± 0.03	0.24 ± 0.04	0.240	1.3	0.24 ± 0.04	0.21 ± 0.05	0.690	0.9	0.18 ± 0.03	0.21 ± 0.05	0.614	1.2
ZT19	Pyruvic acid	0.54 ± 0.03	0.31 ± 0.03	**0.001**	0.6	0.31 ± 0.03	0.42 ± 0.07	0.205	1.4	0.54 ± 0.03	0.42 ± 0.07	0.151	0.8
	Succinic acid	0.68 ± 0.09	1.29 ± 0.25	0.063	1.9	1.29 ± 0.25	1.24 ± 0.16	0.879	1	0.68 ± 0.09	1.24 ± 0.16	**0.022**	1.8
	Fumaric acid	8.46 ± 1.43	6.79 ± 1.09	0.388	0.8	6.79 ± 1.09	8.95 ± 0.68	0.143	1.3	8.46 ± 1.43	8.95 ± 0.68	0.769	1.1
	Malic acid	2.8 ± 0.44	2.44 ± 0.45	0.583	0.9	2.44 ± 0.45	3.21 ± 0.25	0.181	1.3	2.8 ± 0.44	3.21 ± 0.25	0.447	1.1
	α-ketoglutaric acid	0.03 ± 0.01	0.03 ± 0.01	0.888	1	0.03 ± 0.01	0.04 ± 0.01	0.406	1.3	0.03 ± 0.01	0.04 ± 0.01	0.370	1.3
	Citric acid	0.21 ± 0.06	0.22 ± 0.08	0.915	1	0.22 ± 0.08	0.38 ± 0.11	0.295	1.7	0.21 ± 0.06	0.38 ± 0.11	0.237	1.8

Metabolites of the TCA cycle in the liver of rats fed with STD or CAF diet were daily dosage with vehicle or GSPE at night (ZT12) and after 9 weeks were sacrificed at 9 a.m. (ZT1), 3 p.m. (ZT7), 9 p.m. (ZT13) or 3 a.m. (ZT19). The statistically significant p-values ($p < 0.05$) are highlighted in bold.
